# Development of a deregulating microRNA panel for the detection of early relapse in postoperative colorectal cancer patients

**DOI:** 10.1186/s12967-016-0856-2

**Published:** 2016-04-29

**Authors:** I-Ping Yang, Hsiang-Lin Tsai, Zhi-Feng Miao, Ching-Wen Huang, Chao-Hung Kuo, Jeng-Yih Wu, Wen-Ming Wang, Suh-Hang Hank Juo, Jaw-Yuan Wang

**Affiliations:** Department of Genomic Medicine, College of Medicine, Kaohsiung Medical University, Kaohsiung, Taiwan; Department of Nursing, Shu-Zen College of Medicine and Management, Kaohsiung, Taiwan; Division of General Surgery Medicine, Department of Surgery, Kaohsiung Medical University Hospital, Kaohsiung, Taiwan; Graduate Institute of Medicine, College of Medicine, Kaohsiung Medical University, Kaohsiung, Taiwan; Division of Gastroenterology and General Surgery, Department of Surgery, Kaohsiung Medical University Hospital, Kaohsiung Medical University, Kaohsiung, Taiwan; Department of Surgery, Faculty of Medicine, College of Medicine, Kaohsiung Medical University, Kaohsiung, Taiwan; Division of Colorectal Surgery, Department of Surgery, Kaohsiung Medical University Hospital, Kaohsiung Medical University, No. 100 Tzyou First Road, Kaohsiung, 807 Taiwan; Division of Gastroenterology, Department of Internal Medicine, Kaohsiung Medical University Hospital, Kaohsiung, Taiwan; Department of Medicine, Faculty of Medicine, College of Medicine, Kaohsiung Medical University, Kaohsiung, Taiwan; Center for Biomarkers and Biotech Drugs, Kaohsiung Medical University, Kaohsiung, Taiwan; Department of Medical Research, Kaohsiung Medical University Hospital, Kaohsiung, Taiwan; Center for Environmental Medicine, Kaohsiung Medical University, Kaohsiung, Taiwan; Graduate Institute of Clinical Medicine, College of Medicine, Kaohsiung Medical University, Kaohsiung, Taiwan

**Keywords:** Biomarker, Colorectal cancer, Early relapse, miR panel

## Abstract

**Background:**

Colorectal cancer (CRC) is the third leading cause of cancer mortality worldwide and is associated with high recurrence and mortality, despite recent advancements in therapeutic strategies. MicroRNA (miR) deregulation is associated with CRC development and recurrence; therefore, miRs may be reliable biomarkers for detecting early relapse postoperatively.

**Methods:**

In this study ten candidates were identified using miR arrays: miR-7, miR-31, miR-93, miR-141, miR-195, miR-375, miR-429, miR-494, miR-650, and let-7b. Substantial differences were observed in their expression levels between early relapsed (recurrences within 12 months after surgery) and non-early relapsed CRC patients. The validation study, including 50 early relapsed and 54 non-early relapsed patients, confirmed miR expression alterations in cancer tissue samples.

**Results:**

Using a miR real-time quantitative polymerase chain reaction (RT-qPCR), we observed that expression levels of miR-93, miR-195, and let-7b were significantly decreased, whereas those of miR-7, miR-141 and miR-494 showed increases that were more significant in the CRC tissue samples from the early relapsed patients than in those from the non-early relapsed patients. Disease-free survival and overall survival were significantly worse in the high miR-7, miR-141, and miR-494 expression subgroups and the low miR-93 and miR-195 expression subgroups (all *P* < 0.05). A panel of 6 miRs (miR-7, miR-93, miR-195, miR-141, miR-494, and let-7b), at a cut-off value of 2 deregulated miRs, distinguished early relapsed CRC from non-early relapsed CRC, with a sensitivity of 76.6 % and a specificity of 71.4 %. By combining this 6-miRs panel with 6 clinicopathologic factors, at a cut-off value of 4, distinguished early relapsed CRC from non-early relapsed CRC, with a sensitivity of 89.4 % and a specificity of 88.9 %.

**Conclusions:**

This study showed that the developed miR panel has the potential to improve predicting early relapse in CRC patients.

## Background

Colorectal cancer (CRC) is the third leading cause of cancer mortality worldwide and accounts for approximately 608,000 deaths worldwide [1, 2]. Although considerable improvement in radical resection has ensured highly effective treatment of localized diseases, recurrence/metastasis is observed in 25–40 % of patients, leading to death within 5 years of diagnosis [3, 4]. Furthermore, patients’ recurrence period correlates strongly with the patients’ survival period [5]. A reliable biomarker for the detection of postoperative relapse could assist physicians in conducting treatments that are more aggressive and would be beneficial to patients [6, 7]. In our previous researches, we had mentioned the relevant clinicopathological factors which related the early relapse: (1) surgical in resected stage II colorectal cancer is dependent on tumor depth, vascular invasion, postoperative CEA level, and the number of examined lymph node [8]; (2) predictive value of vascular endothelial growth factor overexpression in early relapse of colorectal cancer patients after curative resection [9]; (3) S100B protein expression as an independent predictor of early relapse in UICC stage II and III colon cancer patients after curative resection [10] and (4) predictive factors of early relapse in UICC stage I-III colorectal cancer patients after curative resection [11]. Currently, investigators are searching extensively for the ideal biomarker or indicator for predicting clinical outcomes of CRC patients [7, 12, 13].

CRC tumorigenesis and metastasis involve multistep genomic changes, including the activation of oncogenes, inactivation of tumor suppressor genes, and increases in the ability of cell migration, tissue invasion, and organ colonization [14, 15]. A mature microRNA (miR) is a noncoding small RNA that can regulate the expression of downstream target genes posttranscriptionally [16]. The dysregulation of miRs can be either upregulated or downregulated in cancers and has therefore been suggested to play roles in the carcinogenesis, progression, and metastasis of cancers [6, 16–19]. Consequently, miRs have the potential to serve as biomarkers for cancer detection and prognostic prediction [6, 12, 20].

Although several reports and reviews have suggested that deregulated miRs are involved in the pathogenesis of CRC [17, 21–25], few studies have focused on the association between miRs and the early relapse of CRC. A miR array assay was applied to compare miR profiles between CRC tissue samples (test cohort) from early and non-early relapsed patients. We identified 10 candidates, miR-7, miR-31, mi-93, miR-141, miR-195, miR-375, miR-429, miR-494, miR-650, and let-7b, and validated their role by examining 104 tissue samples (validation cohort). Continual efforts have been made to establish a miR panel to improve the detection of early relapse in CRC patients postoperatively and subsequently to augment therapeutic strategies and clinical outcomes.

## Methods

### Patients and tumor samples

In this study, we recruited 104 patients with primary CRC stages II–III according to the Union for International Cancer Control (UICC) classification (54 nonearly relapsed and 50 early relapsed patients after radical resection) from one institution. All 104 CRC patients who were included into the current study did not receive pre-operative neoadjuvant radiochemotherapy. The definition of radical resection is defined as surgical resection that takes the blood supply and lymph system supplying the organ along with the organ. Postoperative relapse was defined as the occurrence of postoperative local recurrent metastasis (tumor growth restricted to the anastomosis or the region of the primary surgery) or distant metastasis (distant organ metastasis or diffuse peritoneal seeding). Early relapse was defined as postoperative relapse occurring within 1 year after radical resection [5, 11, 26, 27]. No-nearly relapse was defined as postoperative relapse occurring 1 year after radical resection or no relapse until the last follow-up visit. All patients were unrelated ethnic Chinese residing in Taiwan. To determine miRs predicting early relapse, tissues were rapidly frozen in liquid nitrogen after resection. Clinical samples were obtained with informed consent from all patients, and the study protocol was approved by the Kaohsiung Medical University Hospital Institutional Review Board. All patients were followed up until their death or December 2013. The median follow-up time was 29 months (range 5–65 months). Disease-free survival (DFS) was defined as the time between resection and CRC relapse or the last follow-up visit. Overall survival (OS) was defined as the elapsed time between resection and death from any cause or the last follow-up visit.

### RNA extraction and cDNA preparation

Approximately 100 mg of each tissue sample was homogenized using a bench-top homogeniser (Polytron PT1600E; Kinematica AG, Lucerne, Switzerland) in 1 mL of TRIzol reagent (Invitrogen, Carlsbad, CA, USA) for total RNA extraction according to the manufacturer’s instructions. For the miR array, the synthesis of cDNA for the miRs was performed using Megaplex Reverse Transcription Human Pool A and Pool B (Applied Biosystems, Inc., CA, USA). For individual miR assays, the cDNA of each miR was synthesized with a unique primer (Applied Biosystems, Inc.) by using 20 ng of total RNA. For the mRNA quantitative assay, cDNAs were synthesized from 1 μg of total RNA with random hexamers primers by using Reverse Transcriptase (Applied Biosystems, Inc.).

### miR array

The miR array was performed as described in our previous study [27]. In summary, tissue samples from three primary CRC patients (one non-early relapsed and two early relapsed patients) were screened using a miR array (Applied Biosystems, Inc.) containing 667 human miRs and mammalian U6b as the internal control to identify differentially expressed miRs between early and nonearly relapsed CRC patients. A real-time quantitative polymerase chain reaction (RT-qPCR) was performed in the Applied Biosystems 7900HT Real-Time PCR System by using the default thermal cycling conditions of the ABI 7900 Sequence Detection System, Version 2.4 [28].

### Assay for each miR

The TaqMan miR RT-qPCR assay (Applied Biosystems, Inc.) was used to quantify the expression level of each candidate miR. The relative expression level of the miR was normalized to that of the internal control U6b by using the following equation: log_10_ (2^−ΔCt^), where ΔCt = (Ct_miR_−Ct_U6b_). The mean and standard deviation (SD) values of log_10_ (2^−ΔCt^) were calculated.

### Statistical analyses

The continuous variables are represented as mean ± SD, and the dichotomous variables are represented as number and percentage values. Analysis of covariance were performed using JMP Version 10.0 software (SAS Institute, Inc., Cary, NC, USA) and used to compare the mean levels of miR expression between early and nonearly relapsed patients, with other clinicopathologic characteristics as covariates. DFS and OS were calculated using the Kaplan–Meier method, and differences in survival rates were determined using the log-rank test. The linear regression and correlation of the miR RT-qPCR were analyzed. Receiver-operating characteristic (ROC) curves were constructed, with area under the ROC curves (AUC) and corresponding 95 % confidence intervals (CIs) being calculated for each miR. The cut-off value with the highest accuracy (minimal false-negative and false-positive results) was determined. A 2-tailed *P* value less than 0.05 was considered statistically significant.

## Results

### Demographic data

The characteristics of 104 CRC patients (54 non-early relapsed and 50 early relapsed patients) are summarized in Table 1. Their mean age was 69.5 years (range 24–88 years). The status of early relapse is also shown in Table 1. Early relapsed patients had more advanced UICC stages than did non-early relapsed patients (*P* = 0.019, Table 1); however, no significant differences were observed in other parameters including age, sex, tumor depth, or tumor size (all *P* > 0.05). Significant differences were observed in the tumor location (*P* = 0.0001) and the presence of vascular invasion (*P* = 0.003), perineural invasion (*P* = 0.007), and lymph node metastasis (*P* = 0.019) between the non-early relapsed and early relapsed groups.

**Table 1:** Clinicopathologic characteristics of 104 patients with UICC stage II–III colorectal cancer (comprising non-early relapsed and early relapsed patients)

Variables	Non-early relapsed^a^ (N = 54)	Early relapsed^a^ (N = 50)	*P* value
Age (<65/≥65 year)	19/35	23/27	0.261
Gender (F/M)	20/34	20/30	0.756
UICC^a^ stage (II/III)	34/20	20/30	0.019
Depth of invasion (T_1_/T_2_/T_3_/T_4_)	0/2/45/7	0/1/43/6	0.857
Maximum size (<5/≥5 cm)	28/26	25/25	0.850
Location (colon/rectum)	48/6	28/22	0.0001
Vascular invasion (N/Y)	46/8	30/20	0.003
Perineural invasion (N/Y)	46/8	31/19	0.007
Lymph node metastasis [N(−)/N(+)]	34/20	20/30	0.019
Type of tumor (A/M)	52/2	42/8	0.029
Histology (WD/MD/PD)	1/45/8	0/44/6	0.465

	Mean ± SD	Mean ± SD	*P* value
Age	66.50 ± 12.05	65.10 ± 14.43	0.594

*UICC* Union for international cancer control, *A* adenocarcinoma, *M* mucinous carcinoma, *WD* well differentiated, *MD* moderately well differentiated, *PD* poorly differentiated

^a^Early relapse is defined as cancer recurrence within 12 months after surgery

### miR array and follow-up validation

**Fig. 1:**
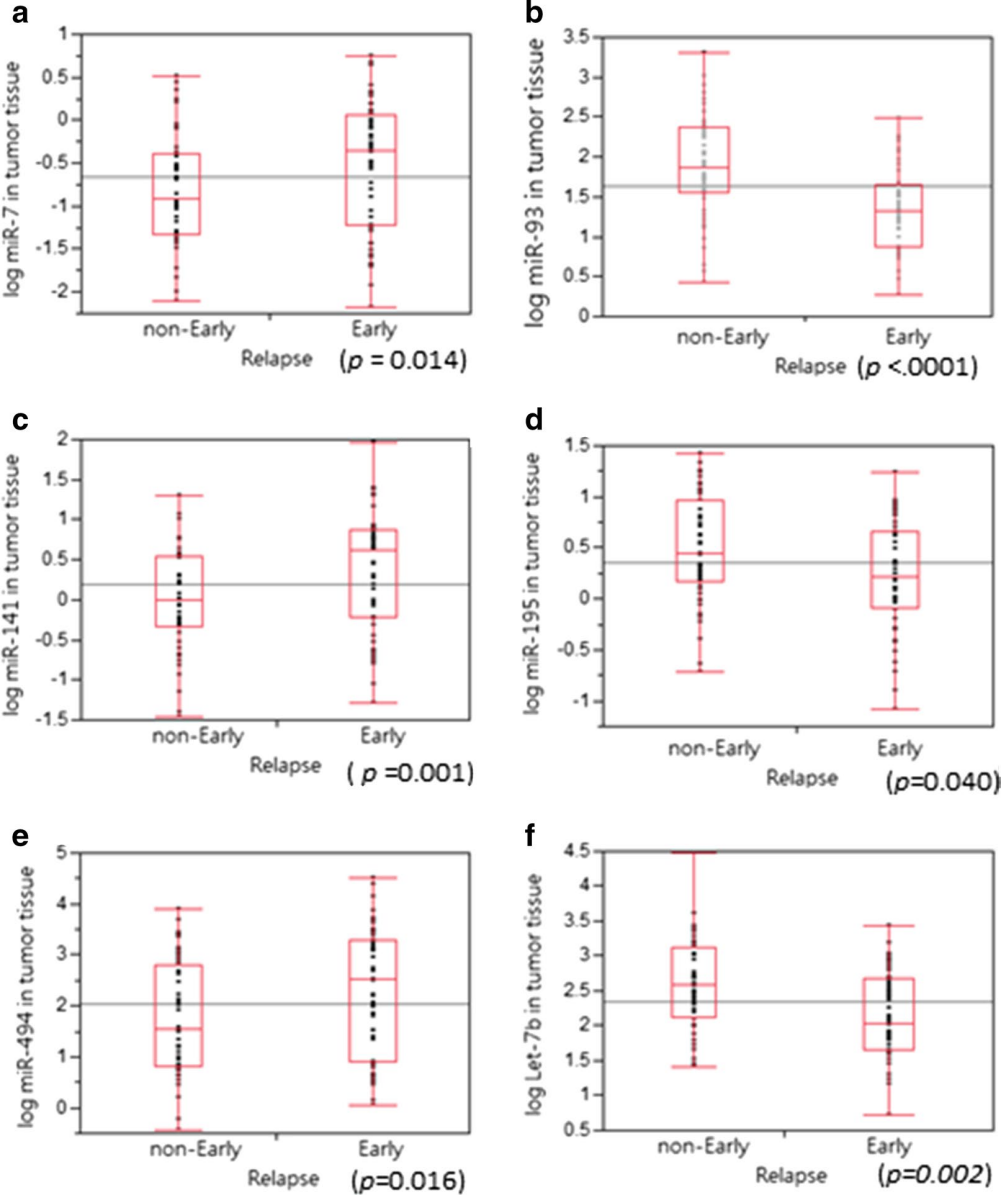
Expression levels of deregulated miR-7, miR-93, miR-141, miR-195, miR-494, and let-7b in human CRC tumors. The relative expression level of miR is represented by log_10_ (2^−ΔCt^); ΔCt = (Ct_miR_−Ct_U6b_), with U6b as the internal control for normalization. **a** The samples from early relapsed patients showed significantly increased miR-7 expression levels (*P* = 0.014). **b** The samples from early relapsed patients showed significantly decreased miR-93 expression levels (*P* < 0.0001). **c** The samples from early relapsed patients showed significantly increased miR-141 expression levels (*P* = 0.001). **d** The samples from early relapsed patients showed significantly decreased miR-195 expression levels (*P* = 0.040). **e** The samples from early relapse patients showed significantly decreased miR-494 expression levels (*P* = 0.016). **f** The samples from early relapsed patients showed significantly decreased let-7b expression levels (*P* = 0.002)

Three primary CRC patients (one nonearly relapsed and two early relapsed patients) were screened using the miR array (Applied Biosystems, Inc.) to identify differentially expressed miRs between early and nonearly relapsed CRC patients (data not shown). We initially used an arbitrary cutoff point of a 2.5-fold change to select 52 potential candidate miRs. Some of them have been reported to be related to colorectal cancers, and their potential target genes also have been checked in silico analysis. We identified 10 miR candidates, miR-7, miR-31, miR-93, miR-141, miR-195, miR-375, miR-429, miR-494, miR-650, and let-7b, and further validated their expression levels between the early and non-early relapsed CRC patients. To confirm the expression levels of the 10 candidates, we examined 104 additional CRC samples: 54 from non-early relapsed patients and 50 from early relapsed patients. Six miRs, miR-195, let-7b, miR-7, miR-93, miR-141, and miR-494, were validated as being associated with early relapse. The expression levels of let-7b, miR-93, and miR-195 were significantly lower in samples from the early relapsed patients (by 0.367-, 0.286-, and 0.608-fold, respectively) than in those from the nonearly relapsed patients (Table 2; Fig. 1). The expression levels of miR-7, miR-141, and miR-494 were considerably higher in samples from the early relapsed patients (by 2.24-, 2.88-, and 3.72-fold, respectively) than in those from the nonearly relapsed patients (Table 2; Fig. 1).

**Table 2:** Expression levels of 10 microRNAs in non-early relapsed and early relapsed colorectal cancer patients

miR	Non-early relapsed (N = 54) mean ± SD	Early relapsed (N = 50) mean ± SD	*P* value
miR-7	−0.843 ± 0.673	−0.493 ± 0.723	0.014
miR-31	−0.054 ± 0.863	0.192 ± 0.816	0.138
miR-93	1.902 ± 0.639	1.359 ± 0.527	<.0001
miR-141	−0.028 ± 0.631	0.432 ± 0.705	0.001
miR-195	0.465 ± 0.561	0.249 ± 0.490	0.040
miR-375	1.781 ± 0.782	1.768 ± 0.781	0.933
miR-429	0.388 ± 0.784	0.335 ± 0.588	0.696
miR-494	1.771 ± 1.093	2.342 ± 1.243	0.016
miR-650	1.358 ± 0.696	1.249 ± 0.766	0.454
let-7b	2.573 ± 0.691	2.138 ± 0.607	0.002

Analysis of the correlations of deregulated miRs with clinical characteristics revealed the association of different miR expression levels with different variables, in addition to a significant correlation with early relapse (Table 3). A high expression level of miR-7 was associated with the presence of perineural invasion (*P* = 0.006). Advanced UICC stage (*P* = 0.029) was associated with a low miR-93 expression level (Table 3). Similarly, an advanced UICC stage (*P* = 0.032) was associated with a high miR-494 expression level (Table 3). In addition, a high expression level of let-7b was observed in younger patients (*P* = 0.005). Among 6 miRs, the expression level of miR-141 and miR-494 were associated with the tumor location (*P* < 0.05, Table 3).

**Table 3:** Correlations of the expression of miR-7, miR-93, miR-141, miR-195, miR-494, and let-7b with the clinicopathologic characteristics of 104 patients with UICC^1^ stage II–III colorectal cancer

Variables	miR-7		miR-93		miR-141		miR-195		miR-494		let-7b	
	Number mean ± SD	*P* value	Number mean ± SD	*P* value	Number mean ± SD	*P* value	Number mean ± SD	*P* value	Number mean ± SD	*P* value	Number mean ± SD	*P* value
*Sex*	61/38	0.900	64/40	0.193	64/39	0.832	63/40	0.235	63/40	0.483	57/38	0.401
Male	−0.66 ± 0.73		1.70 ± 0.66		0.18 ± 0.68		0.41 ± 0.47		1.98 ± 1.18		2.40 ± 0.65	
Female	−0.68 ± 0.70		1.54 ± 0.62		0.21 ± 0.75		0.28 ± 0.63		2.15 ± 1.23		2.27 ± 0.73	
*Age*	60/39	0.427	62/42	0.266	62/41	0.663	62/41	0.074	62/41	0.651	55/40	0.005
≥65 years	−0.71 ± 0.69		1.59 ± 0.70		0.17 ± 0.68		0.29 ± 0.57		2.00 ± 1.28		2.19 ± 0.68	
<65 years	−0.60 ± 0.76		1.72 ± 0.55		0.23 ± 0.47		0.47 ± 0.47		2.11 ± 1.28		2.57 ± 0.62	
*UICC Stage*	51/48	0.053	54/50	0.029	54/49	0.954	50/53	0.056	49/54	0.032	46/49	0.419
II	−0.53 ± 0.69		1.77 ± 0.66		0.19 ± 0.72		0.46 ± 0.57		2.28 ± 1.20		2.40 ± 0.70	
III	−0.81 ± 0.72		1.50 ± 0.61		0.20 ± 0.69		0.26 ± 0.48		1.88 ± 1.14		2.29 ± 0.70	
*Maximum size*	48/51	0.500	51/53	0.856	50/53	0.407	50/53	0.234	50/53	0.400	49/46	0.854
≥5 cm	−0.62 ± 0.74		1.63 ± 0.58		0.13 ± 0.72		0.29 ± 0.59		1.94 ± 1.24		2.34 ± 0.65	
<5 cm	−0.72 ± 0.70		1.65 ± 0.71		0.25 ± 0.69		0.42 ± 0.48		2.14 ± 1.15		2.36 ± 0.72	
*Location*	71/28	0.311	76/28	0.075	75/28	0.023	28/75	0.345	27/76	0.001	27/68	0.602
Colon	−0.72 ± 0.72		1.71 ± 0.65		0.09 ± 0.69		0.39 ± 0.57		1.81 ± 1.14		2.37 ± 0.73	
Rectum	−0.55 ± 0.70		1.46 ± 0.61		0.49 ± 0.68		0.29 ± 0.45		2.69 ± 1.12		2.30 ± 0.56	
*Vascular invasion*	27/72	0.961	28/76	0.117	27/76	0.497	28/75	0.084	28/75	0.881	25/70	0.072
Yes	−0.68 ± 0.78		1.48 ± 0.60		0.28 ± 0.77		0.21 ± 0.53		2.01 ± 1.22		2.16 ± 0.54	
No	−0.67 ± 0.70		1.70 ± 0.66		0.16 ± 0.68		0.42 ± 0.53		2.05 ± 1.20		2.42 ± 0.72	
*Perineural invasion*	26/73	0.006	27/77	0.375	26/77	0.226	27/76	0.457	27/76	0.134	24/71	0.534
Yes	−0.34 ± 0.67		1.55 ± 0.60		0.36 ± 0.84		0.29 ± 0.59		2.35 ± 1.21		2.26 ± 0.82	
No	−0.79 ± 0.70		1.67 ± 0.66		0.13 ± 0.65		0.38 ± 0.52		1.94 ± 1.18		2.38 ± 0.82	
*Lymph node metastasis*	51/48	0.053	54/50	0.029	54/49	0.954	50/53	0.056	49/54	0.032	46/49	0.419
No	−0.53 ± 0.69		1.77 ± 0.66		0.19 ± 0.72		0.46 ± 0.57		2.28 ± 1.20		2.40 ± 0.70	
Yes	−0.81 ± 0.72		1.50 ± 0.61		0.20 ± 0.69		0.26 ± 0.48		1.88 ± 1.14		2.29 ± 0.70	
*Type*	89/10	0.440	94/10	0.350	93/10	0.754	93/10	0.480	94/9	0.627	86/9	0.122
Adenocarcinoma	−0.65 ± 0.72		1.66 ± 0.64		0.18 ± 0.72		0.35 ± 0.54		2.03 ± 1.22		2.39 ± 0.67	
Mucinous carcinoma	−0.83 ± 0.67		1.44 ± 0.71		0.25 ± 0.58		0.47 ± 0.50		2.20 ± 0.96		1.98 ± 0.68	

### DFS and OS analysis

**Fig. 2:**
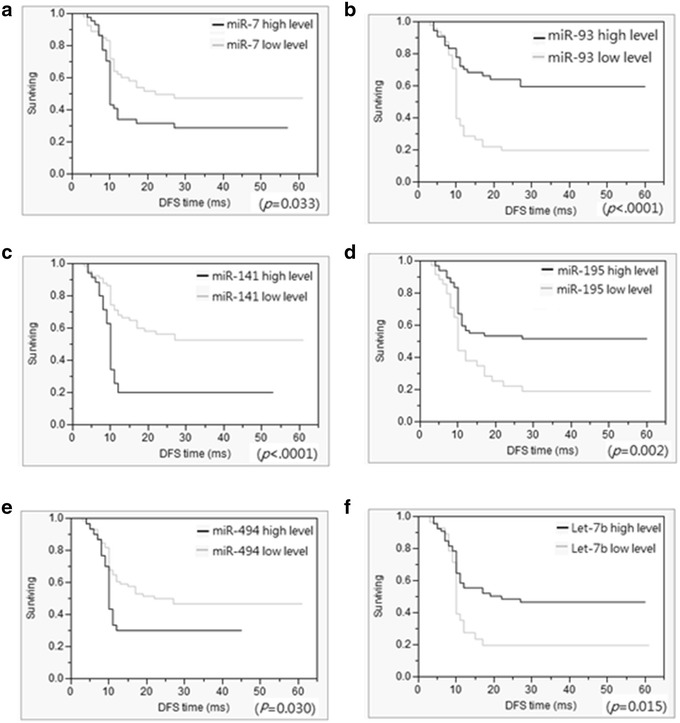
Disease-free survival rates of 104 colorectal cancer (CRC) patients. Disease-free survival rates were assessed using the Kaplan–Meier method, and differences in survival rates were determined using the log-rank test. CRC patients were divided into low and high expression subgroups based on the cut-off value in the ROC curve of miR expression. **a**–**f** Disease-free survival was significantly shorter in CRC patients in the high miR-7 (**a**, *P* = 0.033), miR-141 (**c**, *P* < 0.0001), and miR-494 (**e**, *P* = 0.03) expression subgroups. Disease-free survival was significantly longer in CRC patients in the high miR-93 (**b**, *P* < 0.0001), miR-195 (**c**, *P* = 0.002), and let-7b (**e**, *P* = 0.015) expression subgroups

**Fig. 3:**
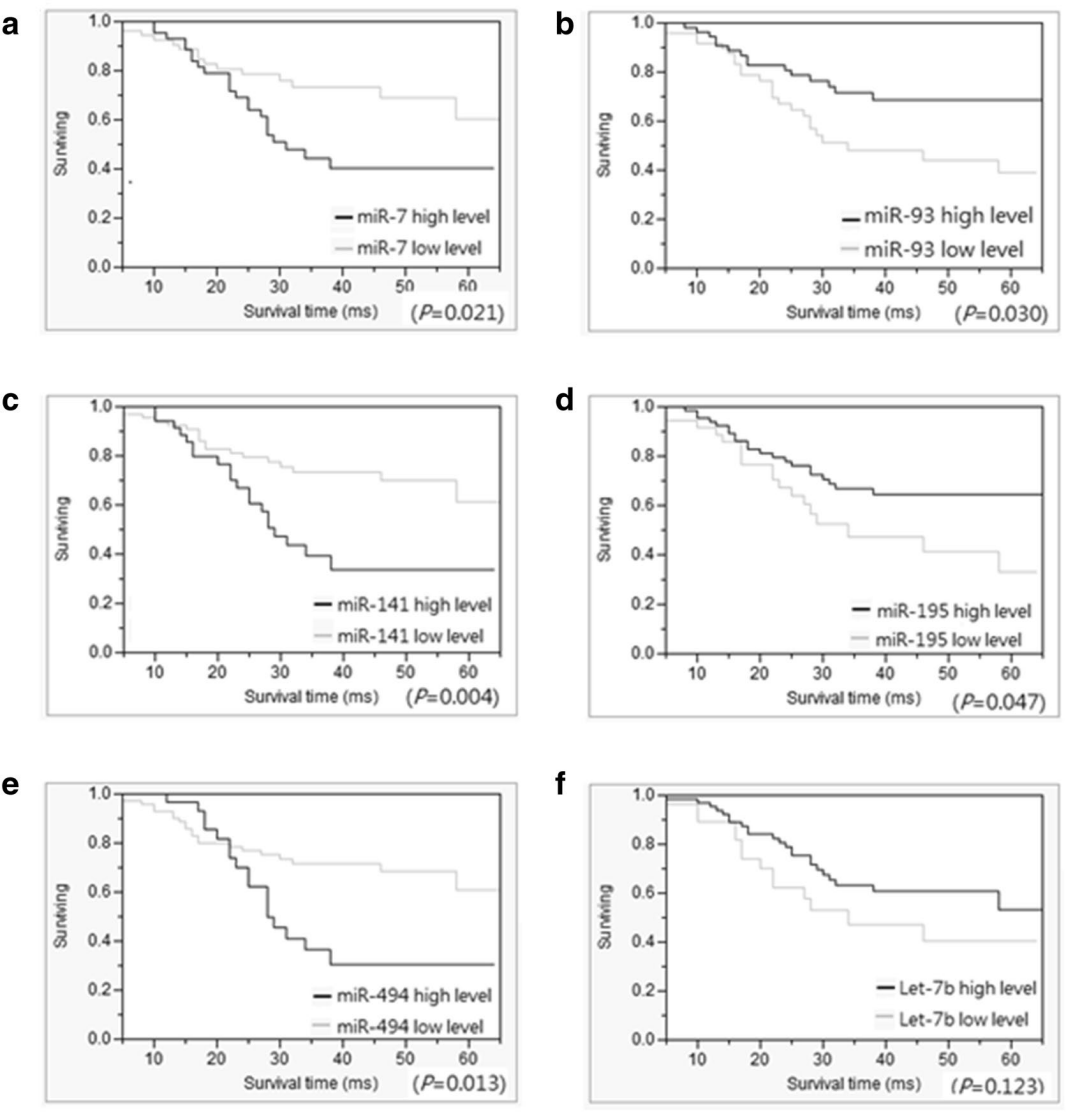
Cumulative survival rates of 104 patients with UICC stage II–III CRC. The overall survival of the CRC patients was assessed using the Kaplan–Meier method, and differences in the survival rates were determined using the log-rank test. Based on the ROC curve of the miR expression level, CRC patients were divided into low and high expression subgroups. (**a**–**e**) Overall survival was significantly worse in CRC patients in the low miR-7 (**a**, *P* = 0.021), miR-141 (**c**, *P* = 0.004), and miR-494 (**e**, *P* = 0.013) expression subgroups and the high miR93 (**b**, *P* = 0.030) and miR-195 (**d**, *P* = 0.047) expression subgroups. **f** No significant differences were observed in let-7B between the high and low expression subgroups (*P* = 0.123)

The CRC patients were divided into subgroups according to the cut-off values in the ROC curve of the miR expression levels: high and low expression subgroups for the corresponding miRs. The DFS and OS of the 104 CRC patients in the two expression subgroups were assessed using the Kaplan–Meier method (Figs. 2, 3). Patients with low expression levels of miR-93 and miR-195 had significantly worse DFS (Fig. 2b, d) and OS (Fig. 3b, d). The patients in the high miR-7, miR-141, and miR-494 expression subgroups had significantly worse DFS (Fig. 2a, c, e) and OS (Fig. 3a, c, e) than did those in the low miR-7, miR-141, and miR-494 expression subgroups.

### ROC curve analysis

**Fig. 4:**
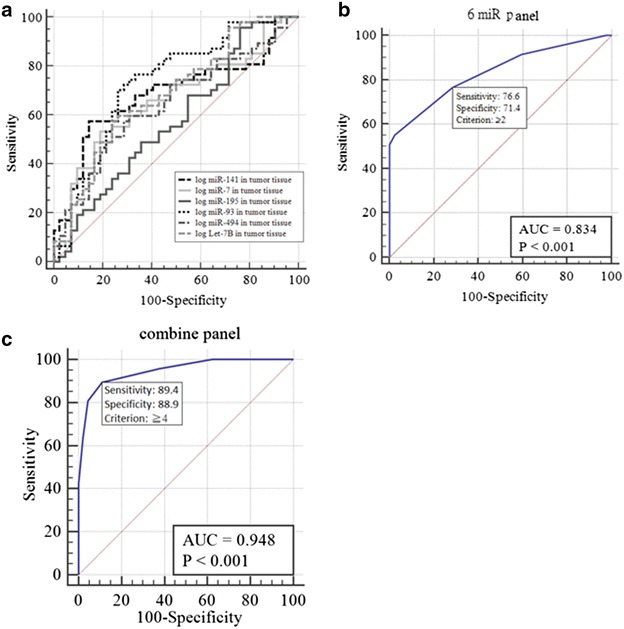
Receiver-operating characteristic (ROC) curves of the six individual miRs and the 6-miR panel of all 104 individuals. **a** ROC curves of the six candidate miRs of all 104 individuals. The sensitivity on the *y-axis* was plotted against the false-positive fraction (1−specificity) on the *x-axis* for various cut-off values. **b** From the ROC plots of miR panel (miR-7, miR-93, miR-141, miR-195, miR-494 and let-7b) expression, two was selected as the cut-off value for detecting early relapsed CRC. The plot is highlighted, with the figures in *parentheses* indicating sensitivity and specificity. The area under the ROC curve is 0.834 (95 % confidence interval = 0.740–0.905). **c** From the ROC plots of combine panel expression, four were selected as the cut-off value for detecting early relapsed CRC. The plot is highlighted, with the figures in parentheses indicating sensitivity and specificity. The area under the ROC curve is 0.948 (95 % confidence interval = 0.881–0.984)

The sensitivity and specificity of the 6 miRs of the CRC patients are listed in Table 4. The diagnostic accuracy of each miR ranged from 56.3 to 73.1 %. ROC curves for each of the six candidate miRs were constructed using data from the 104 individuals evaluated (Fig. 4a). A panel of 6 miRs, with a cut-off value of 2 deregulated miRs, distinguished early relapsed CRC patients from non-early relapsed CRC patients, with an accuracy of 77.4 % (AUC 0.834, 95 % CI = 0.740–0.905); a sensitivity of 76.6 % and a specificity of 71.4 % were obtained (Table 4; Fig. 4b). In Table 1, six clinicopathologic factors (UICC stage, location, type of tumor, vascular invasion, perineural invasion and lymph node metastasis) correlated with relapse status; therefore, we combined 6-miRs panel with these 6 clinicopathologic factors to increase the predictive value (AUC 0.948, 95 % CI 0.881–0.984); a sensitivity of 89.4 %, a specificity of 88.9 % and a accuracy of 89.1 were obtained were obtained (Table 4; Fig. 4c).

**Table 4:** Sensitivity and specificity of the 6 candidate microRNAs of 104 patients with UICC stage II–III colorectal cancer according to real-time quantitative PCR

microRNAs	AUC^a^ (95 % CI)	Cut-off^b^ value	Sensitivity (%)	Specificity (%)	Accuracy^c^ (%)
miR-7	0.647 (0.543–0.739)	−0.37	53.1	80.0	66.7
miR-93	0.752 (0.658–0.832)	1.57	72.0	74.1	73.1
miR-141	0.617 (0.516–0.711)	0.59	76.9	52.6	72.8
miR-195	0.615 (0.514–0.710)	0.93	96.0	26.4	56.3
miR-494	0.634 (0.534–0.727)	3.05	42.9	85.2	61.2
let-7b	0.671 (0.567–0.764)	2.09	55.1	76.1	61.1
6-miR panel	0.834 (0.740–0.905)	2.00	76.6	71.4	77.4
Combined panel	0.948 (0.881–0.984)	4.00	89.4	88.9	89.1

^a^Area under the ROC curve

^b^The optimal cut-off value for each microRNA was calculated by analyzing receiver-operating characteristic (ROC) curves, with the relative expression level of miR being normalized to U6b

^c^Accuracy is defined as the proportion of samples correctly classified into early relapsed (true positives) or nonearly relapsed (true negatives) groups

## Discussion

In the present study, data showed that early relapse was significantly associated with CRC stages that were more advanced, tumor location, and the presence of vascular invasion, perineural invasion, and lymph node metastasis. Initially, we used miR profiling to identify 10 miRs as potential biomarkers for the detection of early CRC relapse. In the validation study, additional samples from CRC patients were analyzed, and we confirmed that early relapse was significantly associated with decreased miR-93, miR-195, and let-7b expression levels and increased miR-7, miR-141, and miR-494 expression levels.

Several previous studies have indicated that miR-93, miR-195, and members of the let-7 family of miRs are downregulated in lung cancer, CRC, and melanoma tissues as compared with normal tissue, suggesting that these miRs are tumor suppressors [29–37]. miR-93 can suppress the proliferation of human colon cancer cells and colon cancer stem cells, with worse OS being associated with low miR-93 expression in CRC patients [37, 38]. miR-195 can suppress tumor cell proliferation and metastasis by targeting several proteins, including BCOX1, BCL-2, and CARMA3 [32–34]. The overexpression of let-7b in vitro can downregulate cell-cycle-related proteins, including cyclin D1, cyclin D3, cyclin-dependent kinase 4, and the high-mobility group protein and oncogene [30, 39]. Tzatsos and Bardeesy found that increased let-7b expression can contribute to a decline in the formation of neurons and the self-renewal potential of neural stem cells during aging [39]. Although let-7b upregulation is observed in aged neural stem cells and is associated with the severity of lens opacity during aging [40], we discovered that downregulation of let-7b is an early relapse biomarker, particularly in elderly patients. miR-141 has been reported to inhibit the migration and proliferation of gastric cancer cells [40, 41], whereas other studies have shown that miR-141 has oncogenic characteristics in CRC and prostate cancer [42, 43]. Yin et al. identified miR-141 as a potential circulating biomarker for metastasis [44], consistent with our findings of high miR-141 expression in early relapsed CRC patients. Previous studies have shown that miR-7 functions as a tumor suppresser in CRC by targeting oncogenic YY1 and XRCC2 [45–47]. By contrast, we found that the upregulation of miR-7 was more frequent in early relapsed CRC patients. Some studies have demonstrated miR-494 as tumor suppressors in CRC [48–51]; however, in this study, miR-494 expression levels showed a more significant increase in the samples from the early relapsed patients than in those from the non-early relapsed patients. Regarding the clinical outcomes, DFS and OS were significantly worse in the high miR-7, miR-141, and miR-494 expression subgroups and the low miR-93 and miR-195 expression subgroups.

There are significant differences in UICC stage, location, type of tumor, vascular invasion, perineural invasion and lymph node metastasis between the non-early relapsed and early relapsed group, these may due to the gene expression profile or miR expression profile difference or tumors with different clinicopathologic characteristics [8, 9, 52–56]. To date, carcinoembryonic antigen (CEA) is the recommended tumor marker for the postoperative surveillance of CRC recurrence in clinical practice. However, in a quantitative meta-analysis of 20 studies (4285 patients), Tan et al. found that the overall sensitivity and specificity of CEA for detecting CRC relapse were 63.9 % (95 % CI 0.613–0.665) and 90.4 % (95 % CI 0.892–0.914), respectively [57]. They concluded that serum CEA is a marker with a high specificity but insufficient sensitivity for detecting CRC recurrence [57]. Su et al. investigated 413 patients and found that the sensitivity of CEA for detecting CRC relapse was 54.4 % and that the sensitivities of CEA for detecting local recurrence, single metastasis, and multiple metastases were 36.6, 66.7, and 75.0 %, respectively [58]. Although it is frequently used for tumor surveillance, the sensitivity of CEA for detecting CRC relapse is not optimal. miRs can be useful biomarkers for detecting CRC relapse. Kanaan et al. and Luo et al. also developed plasma miR panels that specifically distinguished CRC patients from healthy controls [59, 60]. By combining the signature of 6-miRs (miR-7, mi-93, miR-141, miR-195, miR-494, and let-7b) with clinicopathologic characteristics, we successfully detected early relapsed CRC patients with a better accuracy [61]. The sensitivity and specificity of our combine panel for detecting early relapse in CRC patients were improved to be 89.4 and 88.9 %, respectively. The role of the 6-miRs combine panel in the auxiliary role of CEA in the detection of early relapse of post-operative CRC patients might be crucial in the clinical implications. However, a prospective, large-scale study is required to demonstrate the clinical role of this 6-miRs combine panel in the detection of early relapsed CRC patients following radical resection.

## Conclusion

This study is the first to demonstrate that a 6-miR-based biomarker signature consisting of both up- and down-regulated miRs could identify early CRC relapse postoperatively. More prognostic studies should further investigate the association between the dysregulation of these 6 miRs and early relapse to identify CRC patients at a high risk of early relapse after radical resection.
